# Nosocomial infections among COVID-19 patients: an analysis of intensive care unit surveillance data

**DOI:** 10.1186/s13756-021-00988-7

**Published:** 2021-08-12

**Authors:** Clara Chong Hui Ong, Sharifah Farhanah, Kyaw Zaw Linn, Ying Wei Tang, Chu Ying Poon, Allie Yin Lim, Hui Ru Tan, Nur Hafizah Binte Hamed, Xiaowei Huan, Ser Hon Puah, Benjamin C. H. Ho, Margaret M. L. Soon, Brenda S. P. Ang, Shawn Vasoo, Monica Chan, Yee Sin Leo, Oon Tek Ng, Kalisvar Marimuthu

**Affiliations:** 1 IDRTO, National Centre for Infectious Diseases, Singapore, Singapore; 2 NPHEU, National Centre for Infectious Diseases, Singapore, Singapore; 3 Respiratory and Critical Care Medicine, Tan Tock Seng Hospital, Singapore, Singapore; 4 Nursing, National Centre for Infectious Diseases, Singapore, Singapore; 5 Department of Infectious Diseases, Tan Tock Seng Hospital, Singapore, Singapore; 6 Department of Infectious Diseases, National Centre for Infectious Diseases, 16 Jalan Tan Tock Seng, Singapore, 308442 Singapore; 7 Yong Loo Lin School of Medicine, National University of Singapore, Singapore, Singapore; 8 Lee Kong Chian School of Medicine, Nanyang Technological University, Singapore, Singapore; 9 National Centre for Infectious Diseases, Singapore, Singapore

**Keywords:** Healthcare-associated infections (HAI), Device-associated nosocomial infections, COVID-19 outbreak, SARS-CoV-2, Intensive care units (ICU), Catheter-associated urinary tract infection (CAUTI)

## Abstract

Surveillance of nosocomial infections, like catheter-associated urinary tract infection (CAUTI), central line-associated bloodstream infection, possible ventilator-associated pneumonia and secondary bloodstream infections were observed to study the impact of COVID-19 outbreak in ICUs from Tan Tock Seng Hospital and National Centre for Infectious Diseases, Singapore between February and June 2020. Higher nosocomial infection rates were observed in COVID-19 patients, although it was not statistically significant. Moreover, COVID-19 patients seem to be more predisposed to CAUTI despite a higher proportion of non-COVID-19 patients having urinary catheters. Thus, continued vigilance to ensure adherence to IPC measures is needed.

**Supplementary Information:**

The online version contains supplementary material available at 10.1186/s13756-021-00988-7.

## Introduction

With the COVID-19 pandemic continuing globally, the prevention of nosocomial infections is more crucial than ever as secondary nosocomial infections may increase the morbidity and mortality of COVID-19 patients and have been recommended as part of the clinical outcomes to measure for COVID-19 [1–4]. While low levels of T cells were reported among COVID-19 patients [5], suggesting that there is some suppression of the immune system [6], the impact of secondary infections studied is limited. During the pandemic, most healthcare infection and prevention control (IPC) resources are preferentially diverted for outbreak response which may increase the incidence of nosocomial infections among all hospitalized patients [7].

Hence, there is a need to obtain robust data based on established surveillance methods to compare the incidence of nosocomial infections between COVID-19 and non-COVID-19 patients. As part of the outbreak response, we conducted a prospective surveillance for device-associated infections (DAI) and secondary nosocomial bacteraemia in intensive care unit (ICU) using National Healthcare Safety Network (NHSN) criteria. We report the findings from the surveillance in this report.

## Methods

### Study design and setting

We conducted prospective surveillance at the National Centre for Infectious Diseases (NCID), a 330-bedded purpose-built outbreak response facility with 38 ICU beds, and at Tan Tock Seng Hospital (TTSH), a 1700-bedded acute hospital with 72 ICU beds. Outbreak ICUs (n = 2) and medical ICUs wards (n = 2) were included in this surveillance. The surveillance was conducted from 1st February to 30th June 2020 during COVID-19 epidemic in Singapore. Data were extracted from electronic medical records using Research Electronic Data Capture (REDCap) software hosted at National Healthcare Group, Singapore [8]. The Domain Specific Review Board (DSRB) of National Healthcare Group (NHG) provided ethical approval for the study (DSRB: 2020/01242).

### Patient selection and follow up

All patients admitted to the four ICUs were included in the surveillance. Patients who were confirmed to have COVID-19 were managed in the outbreak ICUs and non-COVID-19 patients in medical ICUs.

Patients were reviewed daily by five trained surveillance coordinators from the day of admission to ICU up until two days after they were transferred to the general ward or discharged. For patients with multiple ICU admissions during a single hospitalization episode, only the first ICU episode was included in this analysis. The outcomes of interest were nosocomial infections, including catheter-associated urinary tract infection (CAUTI), central line-associated bloodstream infection (CLABSI), possible ventilator-associated pneumonia (PVAP) and secondary bloodstream infections.

### Data collection and definitions

At baseline, we recorded the age, gender, comorbidities (based on Charlson’s comorbidity index), immunosuppression status (i.e. recent chemotherapy in the last six months, TNF-alpha blocker in the last month, or taking prednisolone 10 mg/day or equivalent steroid on admission), and date of transfer to ICU. Patients were reviewed daily to collect data for presence of invasive devices (i.e. indwelling urinary catheter, central line or endotracheal intubation), microbiological cultures, histopathological tests, radiological investigations and symptoms of nosocomial infections. Data collection form is provided in the Additional file 1. The definitions of device-associated infections, including CAUTI, CLABSI, PVAP and BSI, were standardized according to the criteria provided by the CDC-NHSN [9].

### Statistical methods

We compared the baseline characteristics of COVID-19 and non-COVID-19 patients with Chi-squared tests for categorical variables, and Wilcoxon rank sum test for continuous variables. We calculated the incidence rates (Number of nosocomial infections per 1000 ICU-days or per 1000 device-days) for secondary bacteraemia, CAUTI, CLABSI, and PVAP, and all nosocomial infections (combination of all nosocomial infections under surveillance). We described the monthly trend of nosocomial infections comparing COVID-19 and non-COVID-19 patients in ICUs. Hazard ratios comparing COVID-19 and non-COVID-19 patients were calculated using the Mantel–Haenszel method. A poisson regression model was used to adjust the hazard ratio for the overall nosocomial infections among COVID-19 patients for age, gender, comorbidities, length of stay in ICU, and the presence of invasive devices. The number of events were too small to calculate the adjusted hazard ratio for individual nosocomial infection. All statistical analysis was performed using STATA version 15.0 statistical software.

## Results

A total of 650 patients underwent surveillance for nosocomial infections. Ninety-two patients with incomplete data were excluded (Additional file 1: Figure S1). Of the 558 patients included in the final analysis, 71 were COVID-19 patients and 487 non-COVID-19 patients. Of these, 64.2% were male patients, and the median age of the cohort was 65 years [Interquartile range (IQR): 53–74]. COVID-19 patients were younger, had fewer comorbidities, and stayed longer in ICU (Table 1). COVID-19 patients had indwelling urinary catheters, invasive mechanical ventilation, and central venous lines for a longer duration compared to non-COVID-19 patients (Table 2).

**Table 1:** Distribution of baseline characteristics among COVID-19 patients and non-COVID-19 patients in the ICU

Baseline characteristics	COVID-19 patients (n = 71)	Non-COVID-19 patients (n = 487)	All patients (n = 558)	Chi-squared test
Age, median (IQR)^a^	52 (39–66)	66 (55–75)	65 (53–74)	< 0.001
Male	59 (83.10)	299 (61.40)	358 (64.16)	< 0.001
Length of stay in ICU, Median days (IQR)^a^	6 (2–9)	2 (1–4)	2 (2–4)	< 0.001
*Underlying diseases*				
Overall comorbidities	25 (35.21)	342 (70.23)	367 (65.77)	< 0.001
Chronic pulmonary diseases	2 (2.82)	60 (12.32)	62 (11.11)	0.017
Cardiovascular diseases	4 (5.63)	100 (20.53)	104 (18.64)	0.003
Connective tissue disease	0 (0.00)	11 (2.26)	11 (1.97)	0.201
Peptic ulcer disease	0 (0.00)	13 (2.67)	13 (2.33)	0.164
Chronic neurological diseases	2 (2.82)	79 (16.22)	81 (14.52)	0.003
Chronic kidney disease	5 (7.04)	125 (25.67)	130 (23.30)	0.001
Diabetes Mellitus	19 (26.76)	197 (40.45)	216 (38.71)	0.027
Malignant diseases	1 (1.41)	82 (16.84)	83 (14.87)	0.001
Chronic liver diseases	1 (1.41)	24 (4.93)	25 (4.48)	0.180
Immunosuppression	2 (2.82)	30 (6.16)	32 (5.73)	0.258

^a^Rank sum test

**Table 2:** Incidence rate, hazard ratios, 95% CI and significance tests for various types of nosocomial infections by COVID-19 status

	COVID-19 patients (n = 71)	Non-COVID-19 patients (n = 487)	*P* value
*Nosocomial infection*			
Total ICU-days	727	1847	–
Episodes of nosocomial infection	10^a^	13^b^	–
Incidence of nosocomial infection per 1000 ICU-days (95% CI)	13.76 (7.40–25.56)	7.04 (4.09–12.12)	–
Hazard ratio	1.95 (0.86–4.46)	1	0.105
Adjusted hazard ratio^c^	1.59 (0.60–4.21)	–	0.353
*CAUTI*			
Indwelling urinary catheter	42 (59.15)	354 (72.69)	0.019
Duration of indwelling catheter, Median days (IQR)^d^	6.5 (4–14)	2 (2–4)	< 0.001
Total Catheter-days	555	1443	–
Episodes of CAUTI	4	1	–
Incidence of CAUTI per 1000 catheter-days (95% CI)	7.21 (2.70–19.20)	0.69 (0.10–4.92)	–
Hazard ratio (95% CI)	10.40 (1.16–93.05)	1	0.009
*CLABSI*			
Number of patients on central line	27 (38.03)	191 (39.22)	0.848
Duration of central line, Median Days (IQR)^d^	9 (6–23)	3 (2–7)	< 0.001
Total Central line-days	418	1050	–
Episodes of CLABSI	0	2	–
Incidence of CLABSI per 1000 central line-days (95% CI)	0	1.90 (0.48–7.62)	–
Hazard ratio (95% CI)	–	–	–
*PVAP*			
Invasive mechanical ventilation	27 (38.03)	222 (45.59)	0.231
Duration of mechanical ventilation, median days (IQR)^d^	8 (5–26)	3 (1–5)	< 0.001
Total Ventilator-days	471	1150	–
Episodes of PVAP	1	2	–
Incidence of PVAP per 1000 ventilator-days (95% CI)	2.12 (0.30–15.07)	1.74 (0.43–6.95)	–
Hazard Ratio (95% CI)	1.22 (0.11–13.46)	1	0.87
*Secondary bloodstream infection (BSI)*			
ICU-days	727	1847	–
Episodes of BSI	5	8	–
Incidence of BSI per 1000 ICU-days (95% CI)	6.88 (2.86–16.52)	4.33 (2.17–8.66)	–
Hazard Ratio (95% CI)	1.59 (0.52–4.85)	1	0.413

^a^From the 10 nosocomial infections, a total of 11 organisms were detected. There were 2 (18.2%) *Klebsiella pneumoniae*, 2 (18.2%) *Escherichia coli*, 1 (9.1%) *Enterobacter cloacae*, 2 (18.2%) *Pseudomonas aeruginosa*, 1 (9.1%) *Pseudomonas species*, 1 (9.1%) *Enterococcus faecium*, 1 (9.1%) coagulase-negative *Staphylococcus*, and 1 (9.1%) *Candida species* detected. All enterobacteriaceae and non-fermenters organisms were sensitive to carbapenem

^b^From the 13 nosocomial infections, a total of 17 organisms were detected. There were 4 (23.5%) *Staphylococcus aureus*, 3 (17.7%) *Klebsiella pneumoniae*, 1 (5.9%)* Escherichia coli,* 1 (5.9%) *Enterobacter aerogenes*, 1 (5.9%) *Pseudomonas aeruginosa*, 1 (5.9%) *Enterococcus faecium*, and 6 (35.3%) *Candida species* detected. All enterobacteriaceae and non-fermenters organisms were sensitive to carbapenem. Of the 4 *Staphylococcus Aureus*, 1 was methicillin-resistant

^c^Adjusted for age, gender, comorbidity as a whole, length of stay in ICU, presence of IDC, presence of invasive ventilation, and presence of central line

^d^Rank sum test and only patients with invasive procedures done were included

### Incidence of nosocomial infections

During the study period, 14.8% (10/71) developed nosocomial infections in COVID-19 patients and 2.7% (13/487) of non-COVID-19 patients developed nosocomial infections (Table 2). Out of the 10 nosocomial infections detected in COVID-19 patients, five are CAUTI-related, five with secondary bloodstream infection, one PVAP-related, and none had CLABSI-related infection. Whereas non-COVID-19 patients had one CAUTI-related, eight with secondary bloodstream infection, two PVAP-related, and two had CLABSI-related infection. A total of 11 organisms were detected in COVID-19 nosocomial infections and all enterobacteriaceae and non-fermenters organisms were carbapenem-sensitive. Moreover, of the 17 organisms detected in non-COVID-19 patients, only one out of four was Methicillin-Resistant* S. aureus* while all enterobacteriaceae and non-fermenters organisms were carbapenem-sensitive (Table 2).

The incidence rates of all nosocomial infections among COVID-19 and non-COVID-19 patients were 13.76 per 1000 ICU-days [95% confidence interval (CI), 7.40–25.56] and 7.04 per 1000 ICU-days (95% CI 4.09–12.12) (*P* = 0.11) respectively (Table 2). After adjusting for age, gender, comorbidities, length of stay in ICU, and the presence of devices, there was no significant difference in the hazard ratio for nosocomial infections among COVID-19 patients [adjusted hazard ratio (HR), 1.59; 95% CI 0.60–4.21; *P* = 0.35]. There was a higher incidence of CAUTI among COVID-19 patients and a significant difference in the unadjusted hazard ratio for CAUTI among COVID-19 patients (unadjusted HR, 10.40; 95% CI 1.16–93.50; *P* = 0.01), as the number of events were too small to calculate the adjusted hazard ratio for individual nosocomial infection. However, no difference was observed for CLABSI, PVAP, and secondary bacteraemia.

The monthly rate of nosocomial infection among COVID-19 patients peaked in May 2020 at 22.99 per 1000 ICU-days in COVID-19 patients (Additional file 1: Figure S2). The nosocomial infection rate for non-COVID-19 patients remained relatively stable during the study period. During the study period, the risk of nosocomial infections remained higher among COVID-19 patients compared to non-COVID-19 patients (Additional file 1: Figure S3).

## Discussion

In our study, the nosocomial infection rate in the ICU was noted to be higher among COVID-19 patients compared to non-COVID-19 patients; however, it was not statistically significant. COVID-19 patients seem to be more predisposed to catheter-associated urinary tract infection (CAUTI) potentially due to longer duration of indwelling urinary catheter-days despite a higher proportion of non-COVID-19 patients having urinary catheters. Moreover, our study also reflected a relatively low ICU nosocomial infection (8.5%) compared to other studies [2, 3, 10, 11], which ranges from 7.2 to 46% co-infection in critically ill COVID-19 patients. Nosocomial infections in critically ill COVID-19 patients are also known to have poorer mortality, often requiring intensive care [2, 3, 10, 11]. However, our study was unable to show the rate of mortality amongst patients with secondary infection.

Additionally, on top of having invasive devices, critically ill COVID-19 patients may have increased susceptibility to nosocomial infections due to lymphopenia and reduced immune functions [6]. Studies have observed lymphopenia present in COVID-19 patients, which was also reflected in our preliminary observations in our first few COVID-19 cases in Singapore [5, 12]. Additionally, steroids was not part of standard treatment for acute respiratory distress syndrome in our COVID-19 patients. Furthermore, the COVID-19 virus evades the immune system through the inhibition of interferon type I recognition and signalling, prevents recognition of antigen-presenting plasma and myeloid dendritic cells [6], and undermines lymphocytic activation [13], possibly increasing the hazard for COVID-19 patients to acquire nosocomial infections. However, further studies are warranted to determine if lymphopenia and paresis of other components of the immune response plays a part in nosocomial infections in COVID-19.

Furthermore, the relatively stable nosocomial infection rate in non-COVID-19 patients during the study could also be attributed to heightened infection prevention and control practices during the COVID-19 outbreak. Enhanced precautions, such as improved hand hygiene practices decreases the transmission of nosocomial infections between patients in the ICU. However, changes in care practices such as minimizing contact with suspected or confirmed COVID-19 patients and rostering of available manpower could possibly affect nosocomial infection rates, such as CAUTI in COVID-19 patients as opposed to non-COVID-19 patients. All ICUs in TTSH and NCID are also protocolled to nurse patients in the semirecumbent position unless contraindicated, which reduces the risks of PVAP infection in both patients’ group. Additionally, COVID-19 patients were placed in prone position when medically required. Thus, longer length of stay, use of invasive devices, and reduced immune functions could be potential reasons which resulted in a higher nosocomial infection rate in COVID-19 patients compared to non-COVID-19 patients [7, 14].

Our study has several limitations. First, the small number of nosocomial infections makes it difficult to analyse the risk factors for nosocomial infections and include device-days into our model. Second, we did not audit the adherence to process measures that were in place to prevent nosocomial infections. Hence, we were unable to identify the reasons for CAUTI being the main device-associated infection in COVID-19 patients.

In conclusion, although the incidence of nosocomial infection was not significantly affected by COVID-19 in our centre, continued vigilance to ensure adherence to IPC measures is needed.

## Supplementary Information

**Additional file 1**. Additional figures on recruitment flowchart and monthly nosocomial infection rates; Data collection form.

## References

[1] Marshall JC, Murthy S, Diaz J, Adhikari NK, Angus DC, Arabi YM (2020). A minimal common outcome measure set for COVID-19 clinical research. Lancet Infect Dis.

[2] Bardi T, Pintado V, Gomez-Rojo M, Escudero-Sanchez R, Azzam Lopez A, Diez-Remesal Y (2021). Nosocomial infections associated to COVID-19 in the intensive care unit: clinical characteristics and outcome. Eur J Clin Microbiol Infect Dis.

[3] Baskaran V, Lawrence H, Lansbury LE, Webb K, Safavi S, Zainuddin NI (2021). Co-infection in critically ill patients with COVID-19: an observational cohort study from England. J Med Microbiol.

[4] Cox MJ, Loman N, Bogaert D, O'Grady J (2020). Co-infections: potentially lethal and unexplored in COVID-19. Lancet Microbe.

[5] Xu B, Fan CY, Wang AL, Zou YL, Yu YH, He C (2020). Suppressed T cell-mediated immunity in patients with COVID-19: a clinical retrospective study in Wuhan. China J Infect.

[6] Arunachalam PS, Wimmers F, Mok CKP, Perera RAPM, Scott M, Hagan T (2020). Systems biological assessment of immunity to mild versus severe COVID-19 infection in humans. Science.

[7] Stevens MP, Doll M, Pryor R, Godbout E, Cooper K, Bearman G (2020). Impact of COVID-19 on traditional healthcare-associated infection prevention efforts. Infect Control Hosp Epidemiol.

[8] Harris PA, Taylor R, Minor BL, Elliott V, Fernandez M, O'Neal L (2019). The REDCap consortium: building an international community of software platform partners. J Biomed Inform.

[9] Centers for Disease Control and Prevention (CDC) National Healthcare Safety Network (NHSN) Patient Safety Component Manual 2020. https://www.cdc.gov/nhsn/. Accessed 25 Aug 2020.

[10] Garcia-Vidal C, Sanjuan G, Moreno-García E, Puerta-Alcalde P, Garcia-Pouton N, Chumbita M (2021). Incidence of co-infections and superinfections in hospitalized patients with COVID-19: a retrospective cohort study. Clin Microbiol Infect.

[11] Grasselli G, Scaravilli V, Mangioni D, Scudeller L, Alagna L, Bartoletti M, et al. Hospital-acquired infections in critically ill patients with COVID-19. Chest. 2021;160(2):454–65. 10.1016/j.chest.2021.04.002 PMC8056844 33857475

[12] Young BE, Ong SWX, Kalimuddin S, Low JG, Tan SY, Loh J (2020). Epidemiologic features and clinical course of patients infected with SARS-CoV-2 in Singapore. JAMA.

[13] Chang F-Y, Chen H-C, Chen P-J, Ho M-S, Hsieh S-L, Lin J-C (2020). Immunologic aspects of characteristics, diagnosis, and treatment of coronavirus disease 2019 (COVID-19). J Biomed Sci.

[14] McMullen KM, Smith BA, Rebmann T. Impact of SARS-CoV-2 on hospital acquired infection rates in the United States: predictions and early results. Am J Infect Control. 2020;48(11):1409–11. 10.1016/j.ajic.2020.06.209 PMC7329659 32621857

