# Supplementary material for: Nosocomial infections among COVID-19 patients: an analysis of intensive care unit surveillance data
Source: Antimicrob Resist Infect Control. 2021 Aug 12;10:119. doi: 10.1186/s13756-021-00988-7 (PMC8358905; doi:10.1186/s13756-021-00988-7)
Supplement: Supplementary file 1 — Additional file 1. Additional figures on recruitment flowchart and monthly nosocomial infection rates; Data collection form. [file 13756_2021_988_MOESM1_ESM.docx]

**Supplementary Materials**

**Supplementary Figures**

*Supplementary Figure 1: Recruitment flow chart*
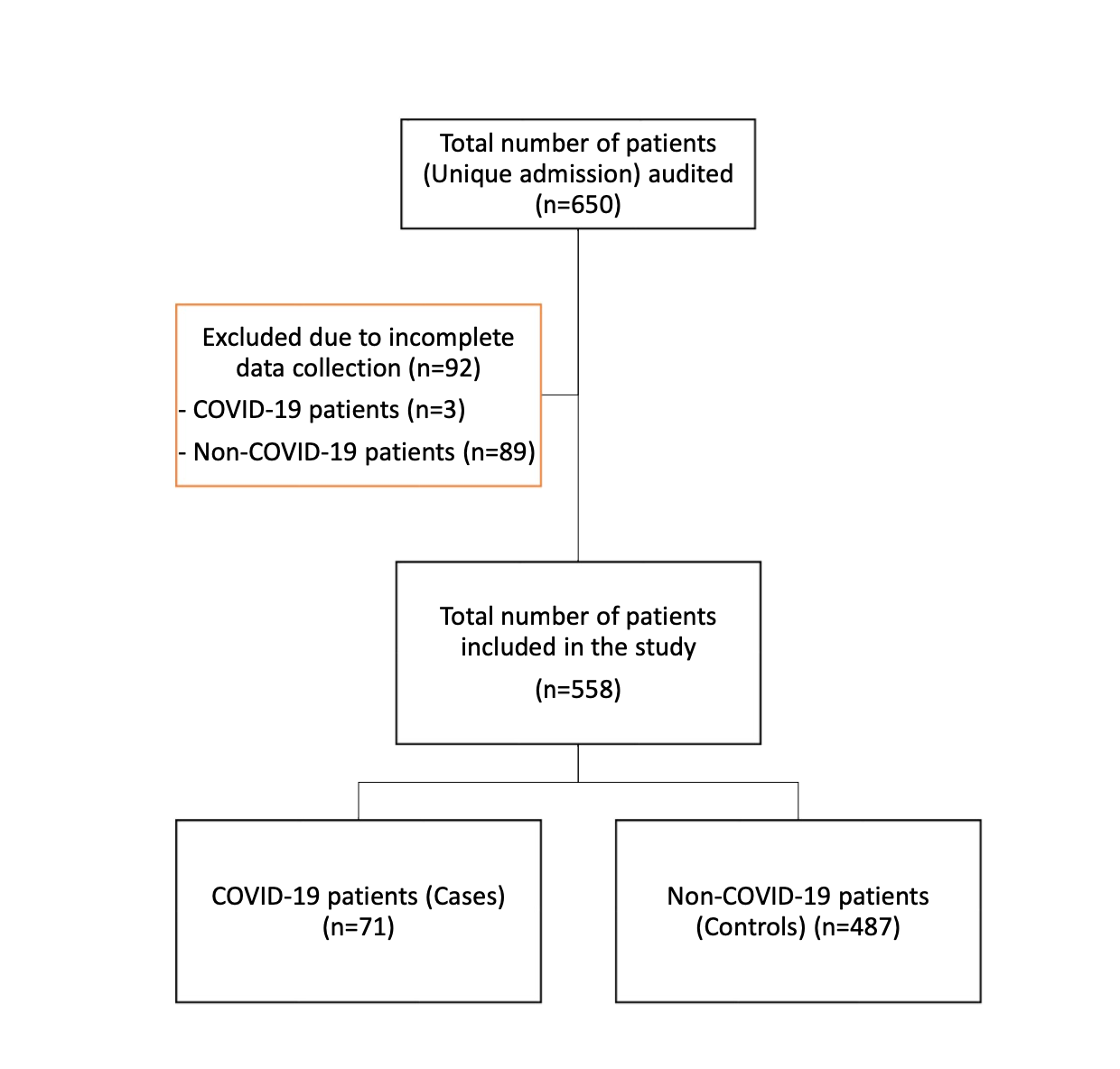


*Supplementary Figure 2. (A) Monthly nosocomial infection rates between COVID-19 and non-COVID-19 patients in ICUs from February 2020 to June 2020, (B) Monthly CAUTI infection Rates between COVID-19 and non-COVID-19 patients, (C) Monthly PVAP infection rates between COVID-19 and non-COVID-19 patients, (D) Monthly CLABSI infection rates between COVID-19 and non-COVID-19 patients and (E) Monthly bloodstream infection rates between COVID-19 and non-COVID-19 patients.*


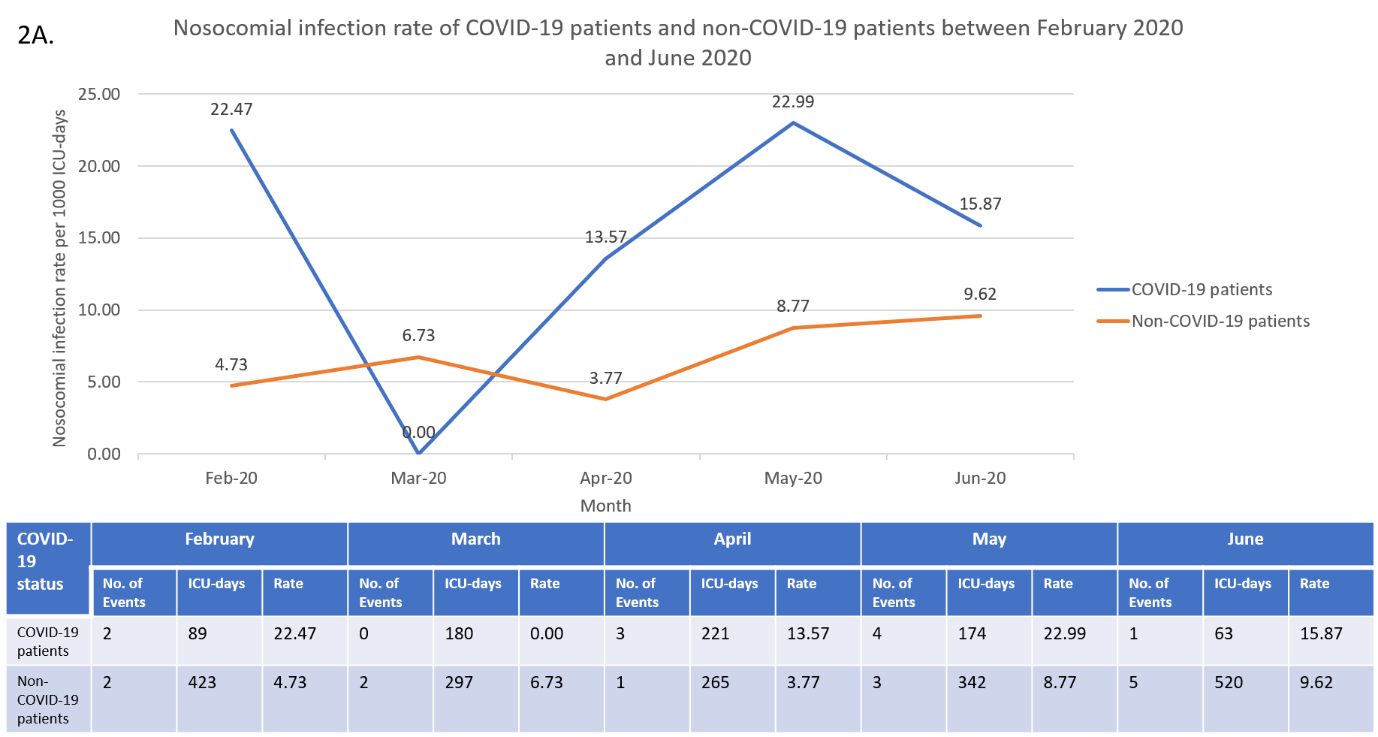

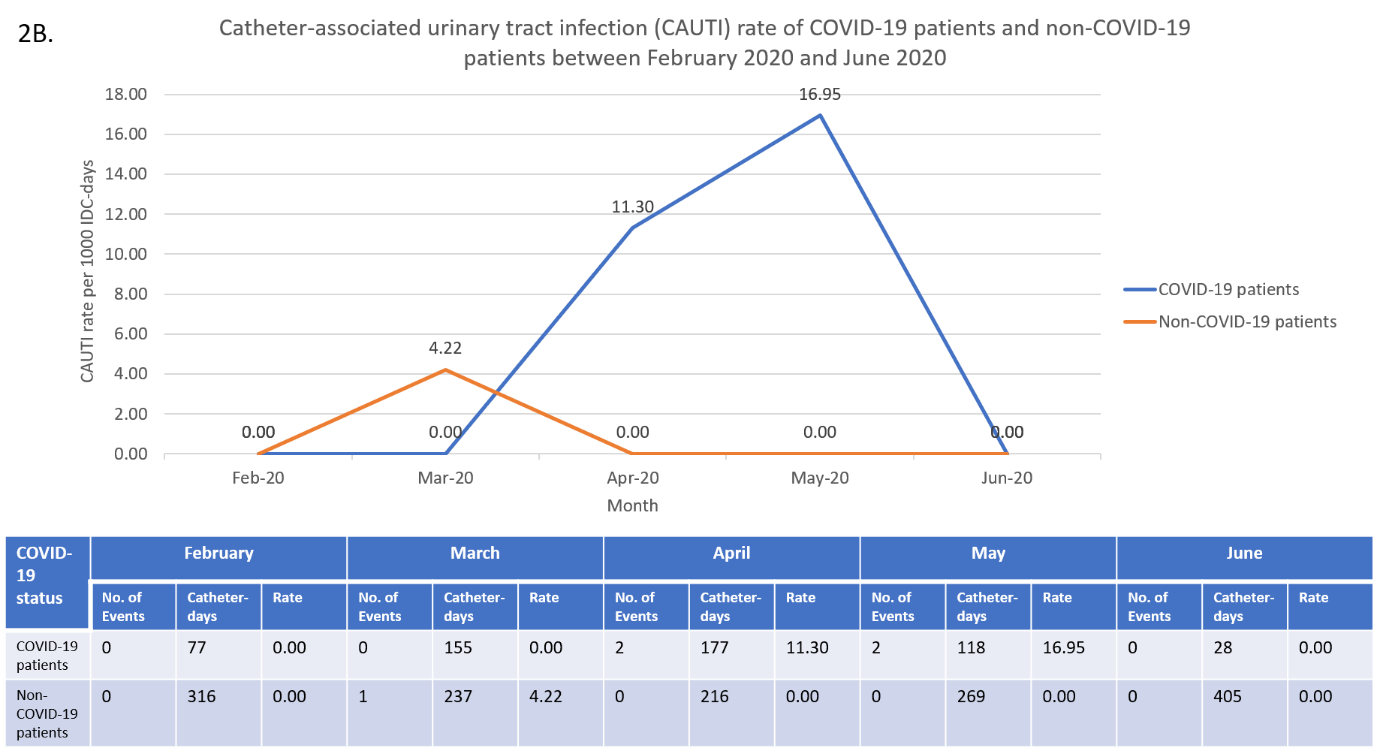

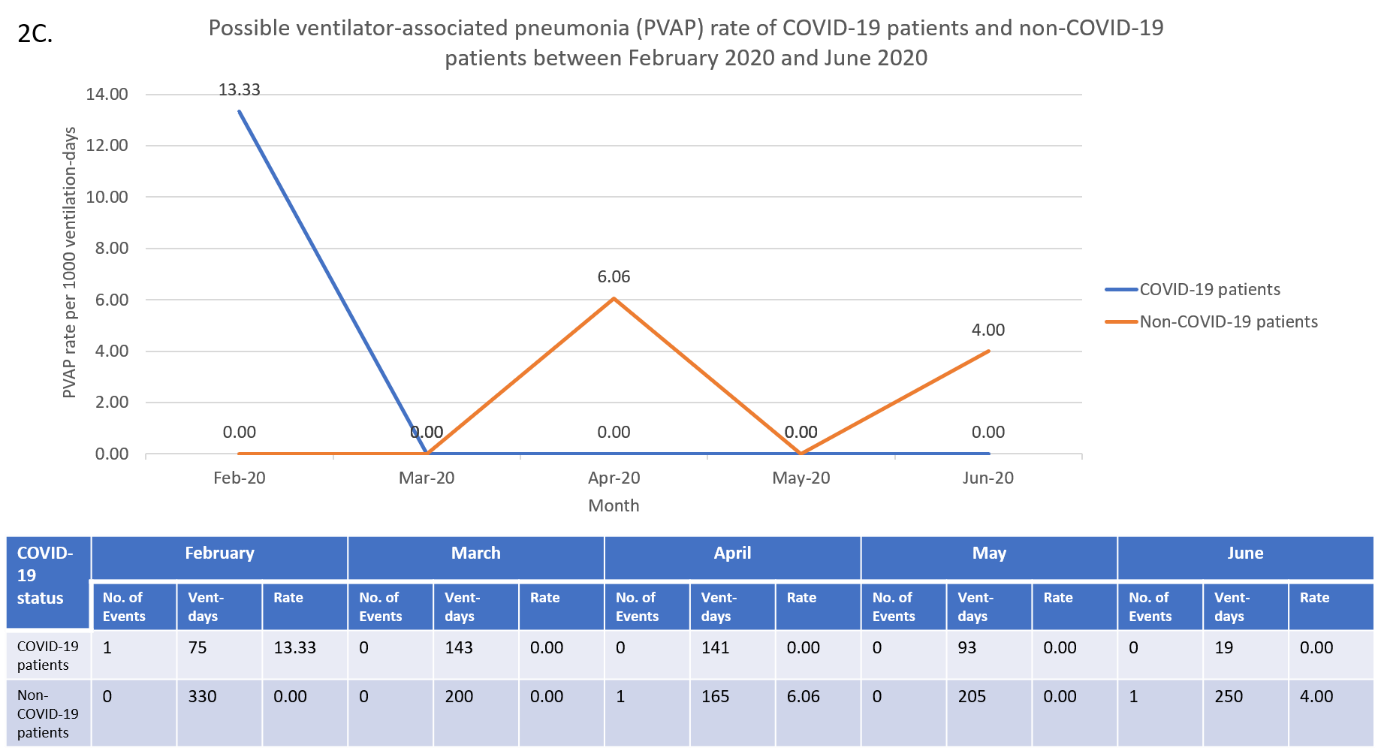

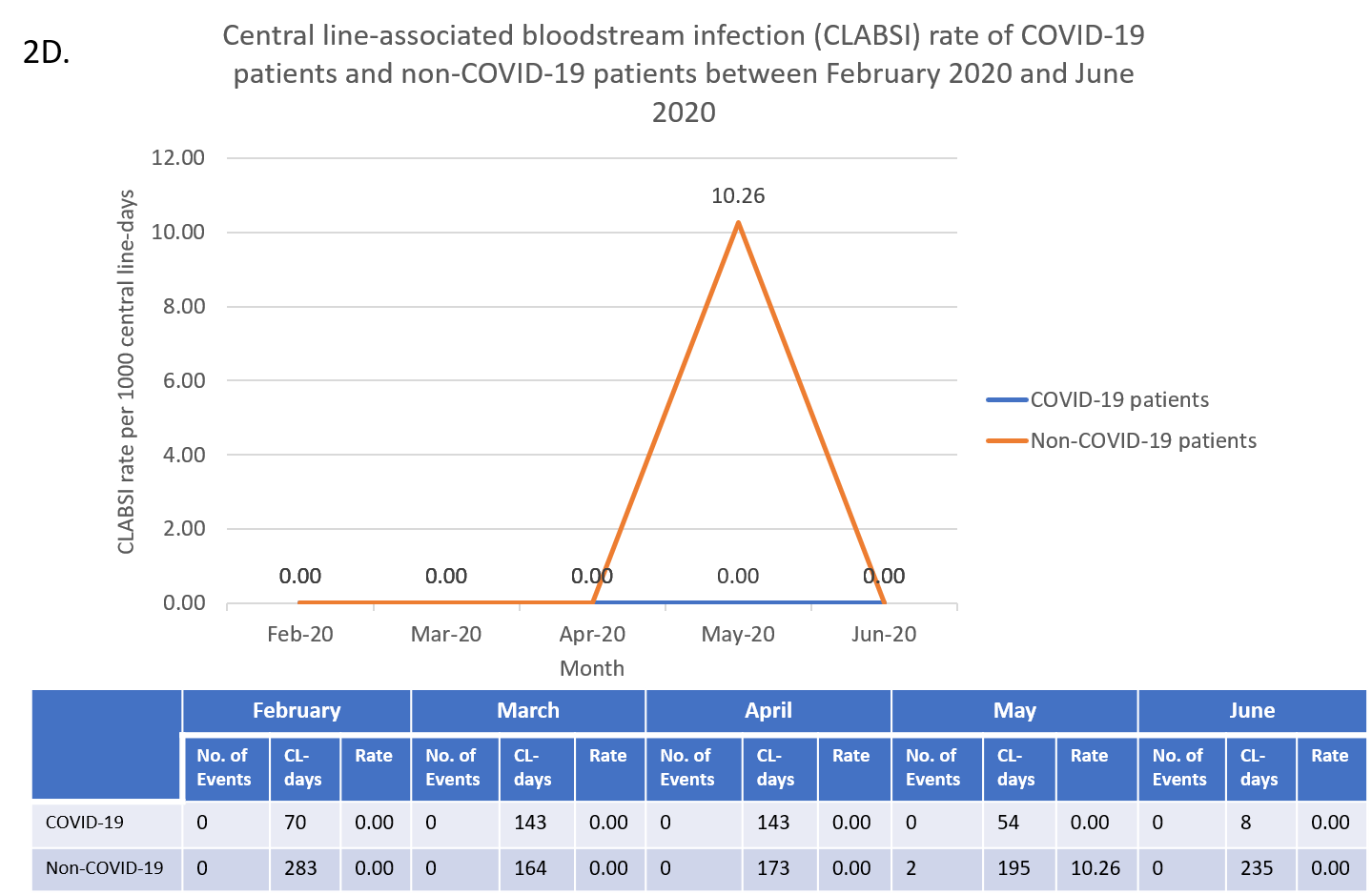

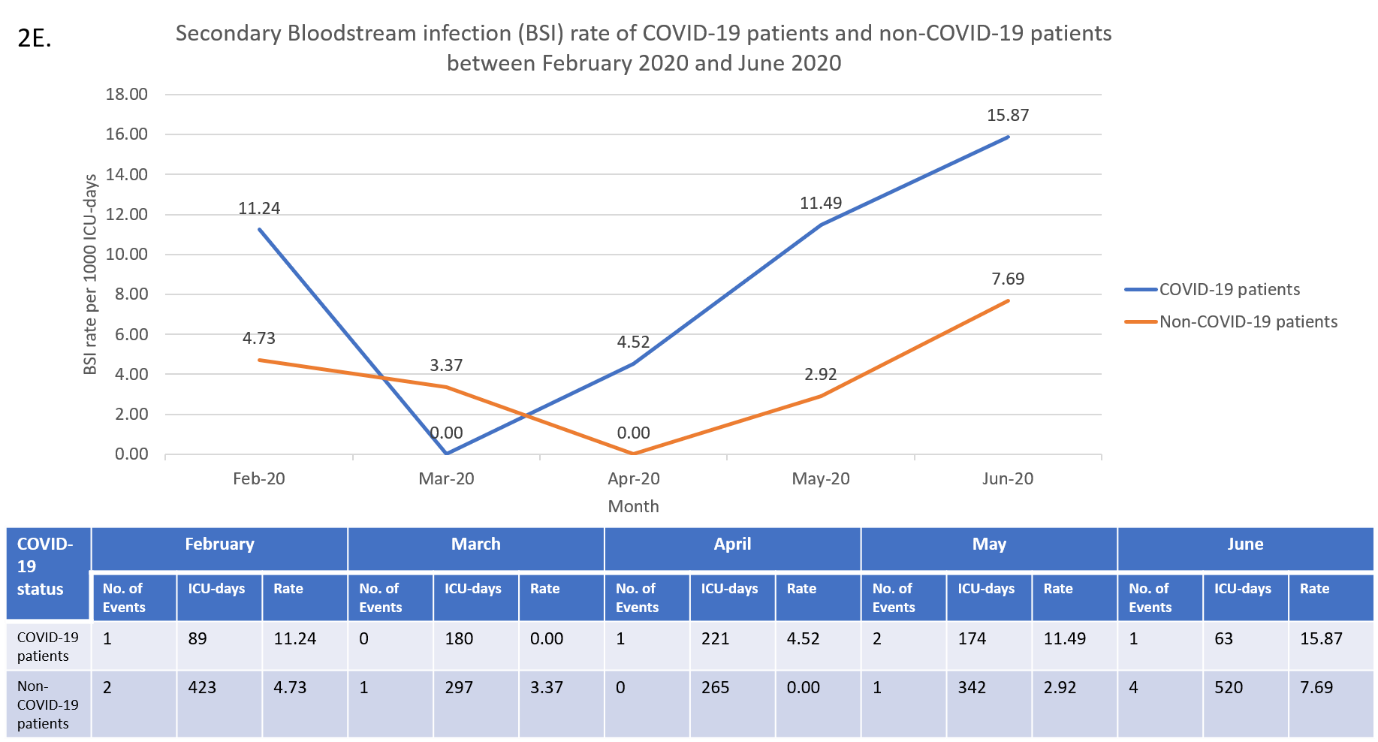


*Supplementary Figure 3: Hazard ratios of nosocomial infection comparing COVID-19 patients with non-COVID-19 patients from February 2020 to June 2020*


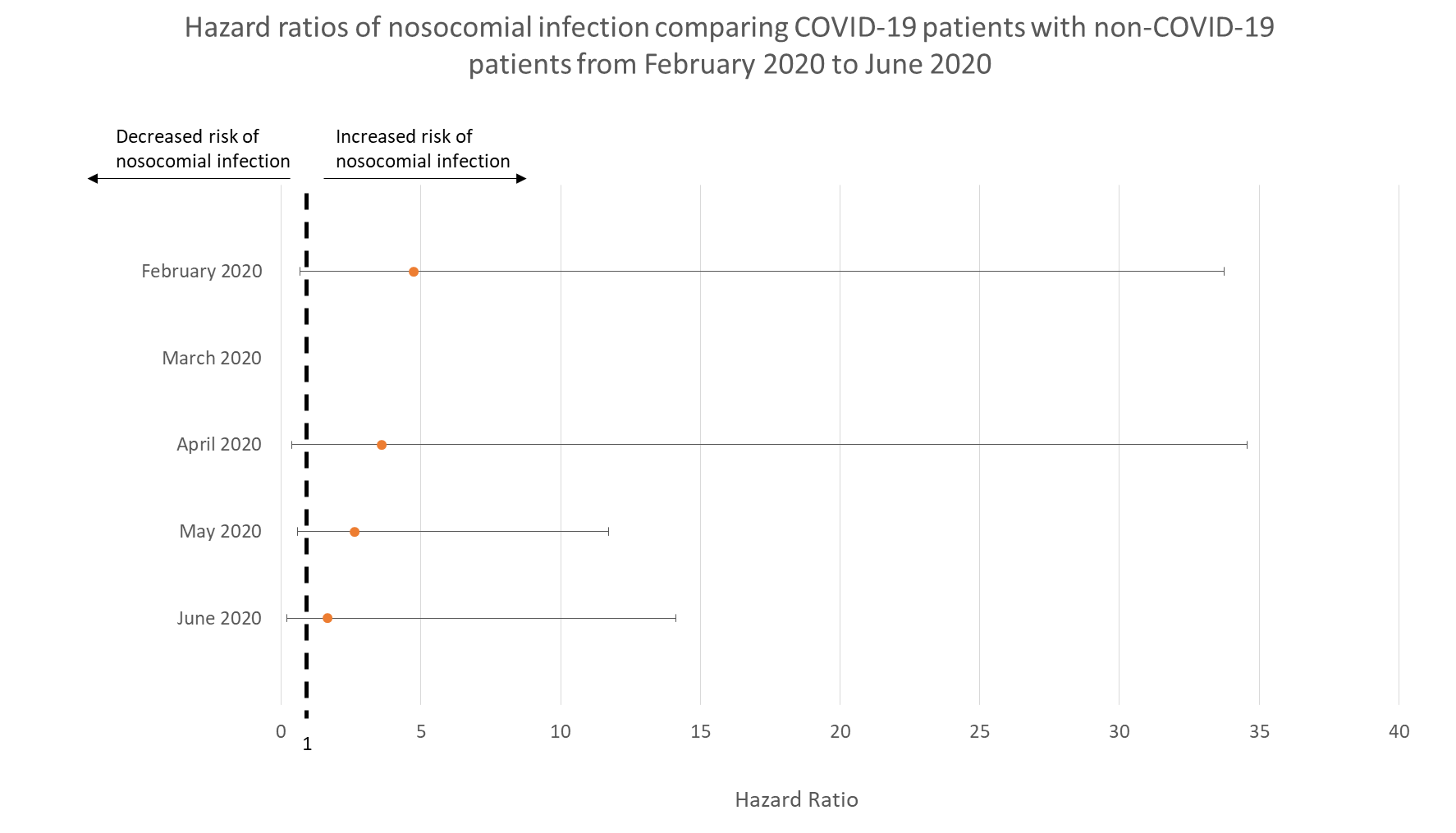


**Nosocomial Infections Surveillance Data Collection Form**

**Baseline Demographics**

REDCap Record ID: __________________________

Date of survey: __________________________

Time of survey: __________________________

Hospital code: __________________________

Ward code: __________________________

Patient code: __________________________

Gender: __________________________

Age: __________________________

Date of admission to ICU: __________________________

Time of admission to ICU: __________________________

Date of discharge from ICU: __________________________

Time of discharge from ICU: __________________________

**Comorbidities**

Chronic pulmonary diseases: __________________________

Congestive cardiac failure: __________________________

Peripheral vascular disease: __________________________

Connective tissue disease: __________________________

Peptic ulcer: __________________________

Cerebrovascular disease: __________________________

Dementia: __________________________

Myocardial infarction: __________________________

Hemiplegia: __________________________

Chronic kidney disease: __________________________

Diabetes Mellitus: __________________________

Solid tumor: __________________________

Haematological malignancy: __________________________

Malignant lymphoma: __________________________

Chronic liver disease: __________________________

HIV: __________________________

Solid organ transplant: __________________________

Stem cell transplant: __________________________

Recent chemotherapy (<6 month): Yes/No

>= Prednisolone 10mg/day or equivalent steroid on admission: Yes/No

TNF-alpha blocker in last month: Yes/No

**At follow-ups**

Day of audit: __________________________

Month of audit: __________________________

Is the patient on IDC at 9AM on [day of audit]-[month of audit]? Yes/No

Is the patient on central line at 9AM on [day of audit]-[month of audit]? Yes/No

Is the patient on invasive ventilation at 9AM on [day of audit]-[month of audit]? : Yes/No

Did the patient meet any definition for nosocomial infection according to NHSN criteria? Yes/No

Specify the type of infection: CAUTI/CLABSI/PVAP/Primary BSI/Secondary BSI

Date of event: __________________________

Type of culture/diagnostic test: __________________________

Date of the culture/test: __________________________

Type of organism(s) isolated from the culture of interest (if applicable): __________________________

Does the patient have at least one of the following signs or symptoms within +/- 3 days of culture date?

- fever (>38.0 C)

- suprapubic tenderness

- costovertebral angle pain or tenderness

- urinary urgency*

- urinary frequency*

- dysuria*

*CANNOT be used if catheter is in place Yes/No

Does the patient have an organism identified from a blood specimen with at least ONE matching bacterium to the bacterium identified in the urine specimen within +/- 3 days of the urine culture? Yes/No

Does the patient have imaging test evidence of urinary system infection +/- 3 days from culture date (which if eqiuvocal is supported by clinical correlation/physician documentation of antimicrobial treatment for urinary system infection)? Yes/No

Does the patient have at least one of the following signs or symptoms within +/- 3 days of culture date?

- fever (>38.0 C)

- localized pain or tenderness Yes/No

Did the patient have an indwelling urinary catheter that had been in place for more than 2 consecutive days in an inpatient location on the date of event AND was either: (Present for any portion of the calendar day on the date of event OR Removed the day before the date of event) Yes/No

Does patient have worsening of oxygenation within +/- 2 days of [date_cul_1]? Yes/No

Please input date of worsening of oxygenation closest to [date_cul_1] and within +/- 2 days: __________________________________

Does the patient have purulent respiratory secretions +/- 2 calendar days from [doe_date_vap]?

Yes/No

Does the patient have the following on or after day 3 of mechanical ventilation AND +/- 2 days from [doe_date_vap]:

- Temperature >38C or < 36C OR WBC count >12,000cells/mm3 or < 4,000 cells/mm3

AND A new antimicrobial agent(s) is started and continued for at least 4 qualified antimicrobial days (QAD) Yes/No

Month of date of event (worsening of oxygenation): ___________________

Are there any types of organisms found in the blood culture? Please record the type organism found: __________________________________

Does the patient have any of the following signs or symptoms within +/- 3 days of the date of culture:

-Fever (>38.0 C)

-Chills

-Hypotension Yes/No

Is the same bacteria identified by a blood culture collected on separate occasions +/- 3 days from current blood culture? Yes/No

Is BSI still a primary BSI after checking for matching organisms from previously diagnosed UTI, PVAP, and BSI as well as checking for secondary bloodstream infection? Yes/No

Is CLABSI still a primary CLABSI after checking for matching organisms from previously diagnosed UTI, PVAP, and BSI as well as checking for secondary bloodstream infection? Yes/No
